# Individual Placement and Support for persons with alcohol and drug addiction in a Swedish context (IPS-ADAS): study protocol for a randomised controlled trial

**DOI:** 10.1186/s13063-024-08007-x

**Published:** 2024-03-27

**Authors:** Ulrika Bejerholm, Anders Håkansson, Marcus Knutagård, Helene Hillborg

**Affiliations:** 1 Department of Health Sciences, Medical Faculty, Lund University, Lund, Sweden; 2 Region Skåne County, Division of Psychiatry, Habilitation and Aids, Lund, Sweden; 3 Department of Clinical Sciences, Medical Faculty, Lund University, Lund, Sweden; 4 Department of Social Sciences, School of Social Sciences, Lund University, Lund, Sweden; 5 Study of Mid-Sweden University, Sundsvall, Sweden; 6 Department of Research and Development, Region Västernorrland County, Sundsvall, Sweden

**Keywords:** Supported employment, Recovery, Mental health, Addiction services, Homelessness, Proactive aging

## Abstract

**Background:**

Employment is a vital source for experiencing well-being and lowering the risk of long-term social marginalisation and poverty. For persons with alcohol and drug addiction, it may also improve sobriety. However, the unemployment situation for this group reflects the knowledge gap in effective interventions to support employment. While Individual Placement and Support (IPS) is recognised as evidence-based supported employment for those with serious mental health problems, no scientific evidence for the target group of addiction exists to date. The aim of the present IPS for Alcohol and Drug Addiction in Sweden (IPS-ADAS) trial is to study whether IPS has an effect on gaining employment for this group.

**Methods:**

The IPS-ADAS trial is a multisite, pragmatic, parallel, and single-blinded, superiority randomised controlled trial (RCT). Participants (*N* = 330) will be randomly assigned (1:1) and participate in IPS plus treatment as usual within Addiction Services (IPS + TAU) or Traditional Vocational Rehabilitation (TVR) available plus TAU (TVR + TAU) for 12 months. The principle of intention-to-treat (ITT) will be applied. The hypothesis is that a significantly larger proportion of IPS + TAU participants will be employed for > 1 day (primary outcome), reach employment sooner, work more hours and longer periods of time, and have a higher income as compared to TVR + TAU participants at 18-month follow-up. We further anticipate that those who benefit from IPS + TAU will use less alcohol and drugs, experience better health, and use less care and support, including support from the justice system, in comparison to TVR + TAU participants, at 6, 12, and 18 months. A supplementary process evaluation, using the IPS Fidelity Scale (25 items) and adhered interviews will address delivery and receipt of the IPS as well as contextual hinders and barriers for coproduction and implementation. Working age (18–65), willingness to work, unemployment, participation in an information meeting about the RCT, treatment for addiction diagnosis, and being financially supported by welfare, constitute eligible criteria.

**Discussion:**

A primary study on the effectiveness of IPS on employment for the new target group of addictions will add to the international IPS knowledge base and inform national policy to include the underrepresented group in working life.

**Trial registration:**

WHO International Clinical Trials Registry Platform ISRCTN10492363. Registered on 14 August 2023.

**Supplementary Information:**

The online version contains supplementary material available at 10.1186/s13063-024-08007-x.

## Background

Alcohol and drug addiction severely contribute to the disease burden (e.g. cardiovascular, sepsis, cancer, suicide, depression, anxiety), and worsen the social and economic situation (e.g. unemployment, homelessness, incarceration, child neglect) [1–3]. In Sweden, there is an inverse relationship between increased use of care and younger age (18-34), and mixed abuse is highest in this group [4]. The young risk long-term social marginalisation, poverty, sickness, and premature death [2, 5]. Hence, addiction severely impacts people’s lives and unemployment is highly prevalent, both nationally and internationally, and the cost of unemployment and sick-leave benefits and the burden for care, school, treatment, and homelessness incentives is huge. The unemployment situation for this group further reflects the knowledge and practice gap of effective interventions to support employment within Addiction Services (AS), internationally [6] and in Sweden [4, 7, 8]. Employment is not being considered a goal in long-term treatment and rehabilitation [9]. If nothing is done, the interrelation between addiction and unemployment continues to foster a downward spiral, since ongoing usage may decrease opportunities to reach and keep employment, and unemployment is likely to increase the use of alcohol and drugs. Effective interventions that both prevent and reduce abuse are critical to develop, study, and implement [1, 10, 11].

Employment has been described as an indispensable tool for transition into recovery for employed individuals in AS treatment [9]. A review further suggests employment as an outcome measure of treatment, but also as a critical ingredient in future innovations within ASs [12]. Employment forms part of society and has shown to be a vital source for experiencing meaningfulness, having economic resources, participating in society, and developing identities [9, 13, 14] and for lowering the risk of long-term social marginalisation and poverty. Work may also improve sobriety, health and well-being and thus support proactive ageing [15]. While the Supported Employment intervention of Individual Placement and Support (IPS) holds a promise [16] and is already recognised in the national policy for AS in Sweden [17], it is yet only an evidence-based intervention for those with serious [18] and moderate mental health problems [18–20]. There is also evidence to suggest that IPS improve health, quality of life, and well-being among those with mental health problems [21–27]. However, no scientific evidence for persons with alcohol and drug addiction exists to date [28, 29], with the exception for a pilot waitlist study [30] and analyses of pooled original data [31], indicating an effect of IPS on employment for the target group.

Previous research shows that context matters and impacts on IPS implementation and thus employment outcomes [16, 32]. Implementation research shows that the primary focus of the Swedish welfare system is on the objective, on organisational constituents rather than on the subjective and experience-based knowledge of users [33–38]. Government-controlled welfare organisations regulate and foster a stepwise rehabilitation chain of fragmented services which is incompatible with the cohesive, integrated, and person-centred approach of IPS. Thus, the uptake may be slow and implementation barriers are critical to assess [16, 25, 33]. Timely, however, a recent national policy for AS addresses the urgency to overcome organisational boundaries to support user goals, a policy that works in favour of the integrated approach of IPS [15].

We acknowledge that the implementation of IPS poses challenges to mindsets and behaviours among professionals within the new field with a tradition of focusing on diagnoses, treatment, disabilities, and at best, stepwise rehabilitation services. We further acknowledge that a trial of complex interventions may be challenging to design, pursue and complete in practice, which may lead to over- and underreported results [39, 40]. Research questions, interventions, and outcomes need to be necessary to the public [41], and interventions more fully described and implemented in practice. To meet this challenge, we suggest coproducing the implementation of IPS and the IPS-ADAS trial within this entirely new field of practice. In the UK, coproduction has long been a policy to mobilise knowledge and innovations in the NHS [42]. It refers to that services need to be built on a knowledge framework where those who receive and deliver them are involved [43–45], which also applies to trial research [46]. Such democratic research may close the gap between the production of knowledge and the actual use of it [47]. A knowledge framework of researchers, professionals, and service users to enhance the implementation and progression of the trial is therefore advocated. Clarity of how coproduction may be evaluated and what level of involvement may be acceptable in a trial context is however lacking but critical to explore [48, 49]. While we want to develop a scientific basis for IPS for the target group, we also want to inform how a sustainable IPS practice in a new field of AS may be built.

## Objectives

The aim of the IPS-ADAS trial is to add to the development of a scientific basis for IPS for the target group of persons with alcohol and drug addiction, with the possibility to influence policy and inform the implementation of an IPS practice in a new field in a Swedish welfare context.

The hypothesis is that a significantly larger proportion of IPS plus treatment as usual (IPS + TAU) participants will be employed for > 1 day at 18-month follow-up (primary outcome), reach employment sooner, work more hours and longer periods of time, and have higher income as compared to traditional vocational rehabilitation (TVR) intervention plus TAU (TVR + TAU) participants among 330 participants. We also anticipate that those who benefit from IPS + TAU will use less alcohol and drugs, experience better health, quality of life and well-being, and use less care and support in comparison to TVR + TAU participants, at 18-month follow-up. A process evaluation, using the Medical Council Framework (MRC) [39] and IPS Fidelity Scale (25 items) [50] and adhered interviews will help to describe the delivery and receipt of the IPS as well as contextual hinders and barriers for coproduction and implementation.

The research questions are:

1. Is IPS + TAU significantly more effective than TVR + TAU at 18-month follow-up in terms of employment (primary outcome), other vocational outcomes, addiction, and care use (secondary outcomes)
2. Is IPS + TAU significantly more effective than TVR + TAU at 18-month follow-up in terms of health, quality of life, and well-being (secondary outcomes)?
3. How and which decisions and processes may be coproduced, what are the benefits and challenges of coproduction, and what mechanisms of coproduction may impact on progress of the trial and the implementation of IPS in a new field?

## Methods

### Trial design

This study protocol presents the IPS-ADAS trial, a multisite, pragmatic, two-arm parallel single-blinded, superiority randomised controlled trial (RCT) of 18 months. Participants (*N* = 330) will by randomly assigned (1:1) and participate in IPS + TAU or TVR + TAU interventions in a Swedish context for 12 months (Table 1). Principle of intention-to-treat (ITT) will be applied and the protocol follows the standards of the 2013 statement of Standard Protocol Items: Recommendations for International Trials (SPIRIT) [51] while the design follows the criteria of randomised controlled of non-pharmacological treatment, the Consolidated Standards of Reporting Trials (CONSORT) [52]. The trial register number is ISRCTN10492363.

**Table 1:**
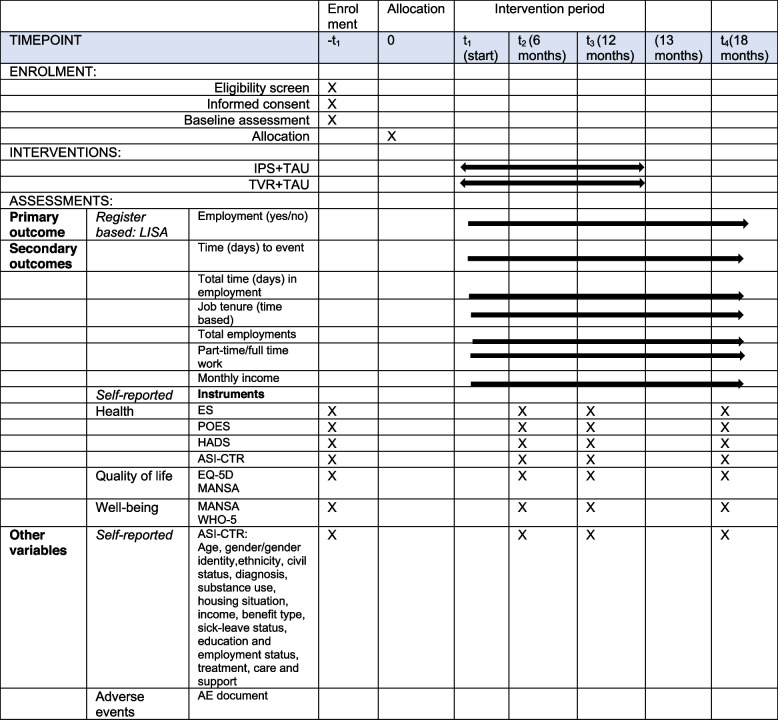
Schedule of enrolment, interventions, and assessment (SPIRIT figure)

*TAU* standard addiction treatment as usual, *IPS* Individual Placement and Support, *TVR* Traditional Vocational Rehabilitation, *ES* Empowerment Scale, *POES* Profiles of Occupational Engagement Scale, *HADS* Hospital Anxiety and Depression scale, *EuropASI-E* E-section of European Addiction Severity Index, *EQ-5D-5L* EuroQoL 5L—health-related quality of life, M*ANSA* Manchester Short Assessment of Quality of Life, *WHO-5* Well-being Scale

### Study setting

The research will be conducted at the Department of Health Sciences at Lund University and involve the Department of Psychiatry, Habilitation and Aids of County Council of Region Skåne. The national research network of the Centre for Evidence-based Psychosocial Interventions (CEPI, www.cepi.lu.se), the international Inter-University Consortium (IUR, www.du.edu/iur), and the user organisation of Verdandi (www.verdandi.se) will be further engaged. Three Addiction Services sites (Lund/Malmö, Stockholm and Södertälje; Municipalities and/or County Council) are included to promote geographical diversity (middle and south of Sweden) as well as locations with various proportions of citizens with foreign backgrounds (Lund 28.6%; Malmö, 46.7%; Stockholm, 34.4%; Södertälje, 57.7%) [4].

### Eligibility criteria and informed consent

Eligibility criteria concern having a working age of 18–65, willingness to work, being unemployed, participation in an IPS-ADAS trial information meeting, being diagnosed with addiction disorder (i.e. alcohol and/or drugs) as a primary diagnosis and in treatment at outpatient AS within the healthcare Region and/or related Municipality, and being financially supported by welfare system (i.e. the Municipality, Public Employment Service or Social Insurance Agency).

Exclusion criteria regard having a lack of interest to become employed and to participate in the IPS-ADAS trial, ongoing abstinence treatment, disability because of another main condition (e.g. serious mental illness, somatic illness), lack of ability to fully engage in IPS due to acute homelessness, being engaged in a lengthy legal process, occupied with plans of suicide, or having had recent attempts (< 3 months).

Eligibility criteria for the sites will concern being an AS provider within the publicly financed healthcare of Region Stockholm and Region Skåne and/or City of Stockholm, Municipality of Södertälje, and Municipality of Lund/Malmö. The trial may be extended to other sites. AS in Sweden are typically divided between Regions and Municipalities and new governmental directives for coproduction and person-centeredness of services are policy [15].

The Employment Specialist is the key professional of the IPS intervention and will have formal IPS training, work experience of IPS, and a position within above stated regions or municipalities.

The AS provider will take informed consent. Initially the professional will provide verbal and written information about the IPS-ADAS trial, including the possible benefits, risks, and alternative interventions. If interested, presumptive participants will be scheduled for an obligatory IPS-ADAS information meeting in a group format or individually. A research administrator, a professional from AS, and a user representative with experienced knowledge of addiction and returning to work will aid presumptive participants to understand the trial more fully, criteria, confidentiality, and the voluntary nature of participation. If criteria are met, the informed consent form may be signed, available on request from the corresponding author.

### Interventions

The comparative intervention will consist of Traditional Vocational Rehabilitation (TVR) services plus Treatment as Usual (TVR + TAU) within AS. We consider TVR the best choice since the service concerns the standard employment support within the Swedish welfare system. The TAU elements of AS treatment regard reducing substance use, increasing health, quality of life, and wellbeing, and securing housing (1–10 h a week). Both the IPS and TVR interventions concern integrating TAU services, including usual care pathways and the use of any medication.

#### The IPS intervention

IPS is guided by an ES who provides IPS to a caseload of < 20 participants for 12 months, with the possibility to abate and refer quality support beyond this point. The time frame is referenced to previous IPS research [22, 28, 29, 53]. The ES works in coproduction with the participant and the IPS network of the AS team (TAU), family and friends (if applicable), and professionals from the Social Insurance Agency (SIA), Public Employment Service (PES), and keep frequent and quality contacts with employers. The professional role of the ES concerns good-quality emphatic counselling according to eight IPS principles (not to be understood or carried out in any particular order): (1) competitive employment as a primary goal, (2) eligibility based on participant’s choice (zero exclusion), (3) rapid job search, (4) integration of IPS with treatment, (5) job search based on personal preferences, (6) on-going support on and off worksite as needed, (7) benefit counselling (SIA/PES) in an early stage, and (8) systematic recruitment and quality engagement with local employers. The dose of delivery is individualised but involves (1) an engagement phase of sharing lived experience and expectations and building a mutual relationship (ES + user), (2) a phase of coproducing and completing a career profile and -plan based on user interest and preferences, (3) a job-seeking and -development phase and if employment is gained, (4) a supported employment phase in which mobilised support strategies of IPS are intertwined. Notably, within the process of IPS support, education efforts are also supported and integrated with the career goal of gaining employment [54–56]. Phases 1 and 2 last for about 1 month, phase 3 until employment is reached, and phase 4 the remaining time. Against the backdrop of previous IPS research [22], the dosage of phases 1, 2, and 3 is approximately 1 h a week, while phase 4 requires 20 min per week. In the Swedish National Guidelines for care and support in cases of substance abuse and addiction, IPS is prioritised as 3 on a 10-point scale, where 1 represents the highest priority [17].

#### The control group intervention

The TVR + TAU intervention will be delivered by professionals within AS and the Municipalities but also from professionals from the Public Employment Service (PES) and the Social Insurance Agency (SIA) throughout the 12-month intervention period. TVR is facilitated through pre-assessment work ability stages according to the institutional logic and regulation of each organisation involved and in connection to regulations of social, sick-leave, and unemployment benefits. In case of sobriety, the first phase 1) involves pre-vocational rehabilitation, including Supported Employment strategies, within labour market units at the municipality (1–5 h per week). If the ability to work across the entire labour market is met, participants may move on to phase 4. If not, users may enter 2 pre-vocational training in day centres (municipality) for engagement in meaningful activities, regulated by law to 5–20 h a week. Phase 3 is vocational training in internship placements (20–40 h a week) through the PES/SIA or municipality, while at the last phase 4, application for employment positions through the PES, for example, may be pursued. In the national guidelines, TVR is prioritised as 6 on a 10-point scale [17]. The TVR intervention resembles an addition to AS treatment (TAU).

### Guidance for discontinuing interventions and promotion of adherence

The criterion for discontinuing or modifying allocated interventions is when participants withdraw consent. In case of worsening symptoms or need of additional support, all participants will receive TAU in addition to IPS and TVR. Thus, IPS employment specialists who are integrated into the treatment team will be noted. Furthermore, the IPS is a person-centred service and is naturally modified in relation to the needs, preferences, and resources of the study participant. The flexible approach with one-to-one communications for imparting and sharing knowledge, scheduled according to the preferences and support needs of the participants affects adherence. IPS further entails ongoing support and outreach activities to maintain contact. Ambivalence, benefits, as well as perceived barriers, are issues discussed throughout the intervention even though study participants are not in an active phase (e.g. adjustment of medication or a dormant period). The coproduced IPS-career profile and plan content will further reinforce, motivate, and encourage participants. Adherence barriers will be addressed in biweekly IPS supervision sessions for employment specialists at each site and at project meetings. Participants in the TVR + TAU intervention will not receive any overall or single guidance, but adherence will be improved and monitored within each step of the rehabilitation process by the relevant welfare actor.

Trial adherence is facilitated by the online RedCap system, providing e-mail reminders to respond to questionnaires. Personal support, including contact and support from a peer, and pen-and-paper options will be available. Preferred contact information and response trajectories will be monitored by research assistants and communicated to the trial management group and operational work groups to enhance assistance and trial adherence of participants.

### Outcomes

Assessment of primary, secondary, and other self-reported outcomes is done at baseline (-*t*^1^), before allocation, and at 6–12–18 months (*t*^1^–*t*^3^) (Table 1). Vocational primary and secondary outcomes will be retrieved retrospectively (> 18 months), via Statistics of Sweden from the LISA register data base (Swedish Longitudinal Integrated Database for Health Insurance and Labour Market Studies) [57]. Secondary and other self-reported variables are reported in an online RedCap system [58]. Adverse Events will be collected continuously.

#### Primary outcome

The primary outcome concerns differences in proportions of gaining competitive employment between the two arms (yes/no, 1 day) at 18 months (*t*^4^), reflecting any employment registered during the trial period. The operationalisation of employment status and time frame are standard in IPS trials internationally and in Sweden [20, 22]. The primary outcome will be collected by Statistics Sweden from the LISA register and be delivered to the trial project team coded. Register data will be validated against users’ self-registration of employment status in the Addiction Severity Index (ASI), and logbooks of employment specialists, by an independent researcher. Self-employment data will also be collected.

#### Secondary outcome

Secondary vocational outcomes are time to event (employment), total time (days) in employment, job tenure (time-based), total employment, proportion of full-time work (40 h/week), and income (amount and benefit type) will be retrieved retrospectively from the LISA register. Secondary outcomes corresponding to health, quality of life, and well-being are descriptive, ordinal, or continuous.

The Empowerment Scale (ES) is chosen to detect changes in perceived empowerment for the benefit of health. It entails 28 statements reflecting five subscales: self-esteem/self-efficacy, power/powerlessness, community activism and autonomy, optimism and control over the future, and righteous anger. Each statement is scored on a 4-point scale ranging from strongly agree [1] to strongly disagree [4], with a total score range of 28–112. Higher scores represent higher levels of empowerment. The Swedish version has shown good internal consistency in previous trials [21, 23].

The Profile of Occupational Engagement Scale (POES) is used to address changes of time use patterns and behaviour as reflected by the engagement level in activities and community life. The first part involves completing a 24-h yesterday time-use diary with 1-h intervals, and a four-column solution corresponding to time, activities, social and geographical environments, and personal reflections about each performance entity. The second part helps to assess the diary content in relation to engagement dimensions according to 9 items on a 4-point scale: daily rhythm of activity and rest, variety and range of occupations, place, social environment, social interplay, interpretation, meaningful occupations, routines, and initiating performance, with a total score range of 9–36. Higher scores are consistent with greater health [59–62]. The POES has sound psychometric properties [61, 62] and has been used to detect change as a primary or secondary outcome measure in previous lifestyle interventions [63–66] and IPS trials [21, 23].

The Hospital Anxiety and Depression Scale (HADS) has 14 items and assists to measure mental health in terms of differences between intervention arms regarding anxiety (7 items) and depression (7 items) [67], also among persons with substance abuse [68]. It is rated on a 4-point scale (0–3 range), and anxiety and depression are scored separately (0 to 21). A higher score indicates a greater level of anxiety and depression, and cut-off scores are available [69]. HADS is used in general medical settings, including occupational health, with sound psychometric properties [70].

The Assessment Severity Index (ASI) is widely used to address the severity of substance use, health and social problems and the need of treatment. ASI holds 7 key domains: medical status, employment and support, drug and alcohol use, legal status, social and psychiatric status [71, 72], and has sound psychometric properties [73]. In the present IPS-ADAS trial, the ASI interview questions are replied online (RedCap), with support from the research administrator if needed. Variables relevant to sociodemographic characteristics, substance abuse and care and support are particularly of interest for the present study (i.e. age, gender/gender identity, ethnicity, civil status, diagnosis, substance use, housing situation, income, benefit type, sick-leave status, education and employment status, treatment, care, and support). A composite score may be summarised for ASI, but the rationale for using the ASI questions in the trial is to benefit from the context-specific index developed for the target group within AS.

Quality of life (QoL) outcomes are assessed by the EuroQol 5 Dimension (EQ-5D) [74] and the Manchester Short Assessment of Quality of Life (MANSA) instrument (see below) [75]. The EQ-5D is used to study the effectiveness of perceived QoL dimensions. It comprises 5 health-related quality of life dimensions, mobility, self-care, usual activities, pain/discomfort, and anxiety/depression with three gravity levels (no problem, moderate problem, and extreme problem), and a visual analogue scale on global QoL (1–100) [74]. An economic evaluation is also available, i.e. the scoring of dimensions helps to quantify 243 health states that can be converted to an index utility score and further translated according to country-specific tariffs which value may be considered as the quality-adjusted life-years (QALY). EQ-5D is well known to assess health interventions, also IPS [24].

MANSA is a brief QoL measure developed for persons with mental health problems [75] and has been used in previous IPS research to detect differences between intervention arms [21–23]. MANSA has 12 items on satisfaction with: life as a whole (global well-being), work situation, finances, friends, leisure, living and housing situation, safety, fellow residents, sexual life, family relations, and physical and mental health. Satisfaction is rated on a 7-point scale, with a sum-score range of 12–84. Higher scores indicate better QoL. MANSA has sound psychometric properties and is widely used [75, 76].

WHO-5 Well-being index (WHO-5) is a brief measure of well-being of the last 2 weeks and consists of five positively worded questions to be rated on a 6-point rating scale. The raw score is transformed to a score between 0 and 100 where a higher score indicates worse well-being. The well-being index was developed in 1998 and has been validated as a measurement instrument in a number of studies [77].

To conclude and improve fidelity to the IPS intervention the Supported Employment Fidelity Scale (SEFS) [50] will be used at 6 and 12 months to conclude and detect changes in service delivery, receipt, and fidelity. Data is collected by the process leader (each site) and research administrator the preceding month. Several data sources, e.g. interviews with IPS participants, unit managers, processors, IPS employment specialists and managers, IPS documentation and statistics will form the basis for the review. This standardised procedure of monitoring adherence entails scoring (1 to 5) of 25 items related to staffing, organisation, and services. Higher scores indicate greater adherence to IPS and a higher expectation of vocational outcomes. An external IPS expert will review the gathered data and be in dialogue with the process leader at each site. Adherence to IPS will be shared in project- and operational work groups as well as with a steering group of integrated actors in which context strategies will be developed to target improvements. The TVR intervention will be reviewed in relation to the SEFS scale as well since no available TVR fidelity scale exists. This allows for comparisons between groups regarding similarities and diverse features [20, 22].

### Participant timeline

**Fig. 1:**
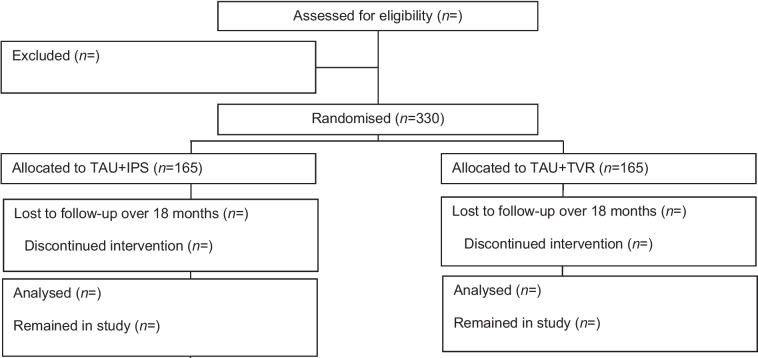
Trial Figure of the IPS-ADAS trial

Enrolment begins when presumptive study participants gain brief written and verbal information about the IPS-ADAS trial from professionals within the AS (Fig. 1). Once inclusion and exclusion criteria are broadly met (pre-screening), interested persons are invited to partake in an information/dialogue meeting (group/individually) to understand eligibility and the meaning of participating in the trial more fully, before signing their written consent (30–45 min). Their written consent may also be handed in afterwards. In connection to enrolment, baseline data is contained (questionnaire battery, 30–50 min) after which allocation to one of the two interventions (IPS + TAU and TVR + TAU) is performed. Participants will attend the interventions for 12 months. The questionnaire battery is repeated at 6 (168 days), 12 (365 days) and 18 months after the randomisation date, with a 21-day window after the due date at the 6- and 12-month follow-up, and 28 days at 18 months.

### Sample size

The primary outcome is used to calculate the required sample size to detect a significant mean difference between the intervention groups at 18 months follow-up (employment rate, yes/no). No previous trial has been published within the field of AS, why the estimation is done against previous IPS trials. According to Marsden et al. [28], using a DELTA^2^ guidance based a meta-analysis from 2019 [18], 302 participants were deemed to be a sufficient sample size (95% CI). When departing in previous Swedish high-fidelity IPS trials, targeting persons with psychosis [20] and depression [22], power calculations (Fisher’s exact test) show the required sample size of 60 and 40 respectively to detect a power of 80% and alpha level at 0.05 (95% CI). One point of departure for aiming at a larger sample size than previous trials is that the dropout rate for persons in AS is assessed to be equivalent or even larger than for those with mental health problems, according to the attendance rates in similar interventions within AS in one of our trial contexts (City of Stockholm). A rate of ≥ 30% drop-out must therefore be considered. Another point of departure regards the criticality to detect statistical a mean difference for secondary outcomes (e.g. ordinal data) which according to a meta-study needs to increase in IPS trials, overall [78]. Lastly, the employment specialists typically have a caseload of a maximum of 20. But since their capacity to support persons with addiction is unknown, the number of maximum participants we estimate to be 15. In all, we intend to involve 9 employment specialists (Stockholm, *n* = 5; Södertälje, *n* = 3, Lund/Malmö, *n* = 3). As a suggestion, the ambition is to include a total of approx. 165 + 165 participants, a total of 330, Stockholm (*n* = 150) and Södertälje (*n* = 90) (East Middle Sweden) and Malmö/Lund (*n* = 90) (Southern Sweden). Distribution of the participants between the locations may be adjusted in accordance with recruitment rates at each site.

### Recruitment, allocation, and blinding

For each site, enrolment will last for about 18 months, from September 2023 to February 2025. Healthcare professionals from AS will assist to identify eligible participants by reviewing health records and approaching users and screening will last until the population of the target is reached for each site. Verbal information will further be advertised in waiting rooms and on social media of research- and user organisations targeting the group of interest. In connection with this initial recruitment phase, it will also be possible for presumptive users to self-register and pose their interest at a website whereupon they are referred to healthcare professionals for brief oral and written information about eligibility.

If criteria are met, presumptive users will be invited to partake in an information meeting (group or individually) at their site. The meeting will target the interventions as well as issues around the RCT design, randomisation, and ethical approval. The content and delivery of the information meetings will follow a premade protocol description and meetings will be held by a healthcare professional and researchers, a research administrator, and a user representative with lived experience of addiction and re-entering employment. Family and friends of presumptive participants may also partake for support. The aim is to provide democratic spaces to inform eligible users and openly discuss the project. Individual meetings may be held by a healthcare professional or a researcher. Any time after attending the meeting users may hand in their written consent. The duration of the recruitment will be approximately 1 year for each site respectively.

After completion of baseline assessment, participants will be randomly assigned to one of the two intervention arms (ratio 1:1) using randomly varying block sizes, using a randomisation schedule automatically generated in RedCap to assure allocation concealment, with no stratification. A randomization list (Excel file) will be kept separate and be administrated by a research administrator who will manage the allocation sequence, enrollment of participants, and assignment to interventions by sending a standardised letter about the IPS + TAU and TVR + TAU intervention respectively by mail, within a week.

The trial will be single- and assessor-blinded, i.e. researchers will have no knowledge of the identity or allocation of users. It will not be possible to mask the allocation status for those receiving or delivering the interventions. Participants will be informed not to reveal their allocation status to research assistants who will be blinded. Once data collection has been completed the independent research assistant will code the allocation status (As and Bs) to ensure blinding throughout analysis and conclusion of results.

### Data collection and management

Researchers will retrieve anonymised primary and secondary vocational, income and benefit data from the LISA database in which data from the labour market, social and educational sector is integrated and updated annually, focusing on the individual as the primary object. Data will be retrieved by Statistics Sweden and the procedure is that the research assistant will submit personal security number data with a variable order to Statistics Sweden which they co-run and deliver back to the researchers pseudonymised. In addition, vocational data will be registered by the participants in connection to RedCap data collection (ASI).

Baseline and follow-up data will be collected by means of questionnaires online via RedCap (Research Electronic Data Capture) [58] and will take about 30–45 min to complete. Data collection on clinical, social, and demographic characteristics, will be obtained by the ASI, while secondary outcomes on health, quality of life and well-being by psychometrically sound self-reported instruments tested within the field and assist to increase the external validity of the trial [21–24, 28, 29]. Instruments are further detailed in the Outcomes section. Data collection will be administered via Lund University and the online survey platform for clinical trials (REDCap) [58] at − *t*^1^, *t*^1^–*t*^4^ (see Table 1) and include all participants unless they withdraw. They will receive an e-mail with a link to the survey and respond to the questions online, or if they prefer a pen-and-paper format. They may also ask for personal assistance by the research assistant or peer support, both having received 1-day education about concepts, psychometry, and interview training by the trial management group. Either option, responses are registered in the RedCap system (Research Electronic Data Capture) hosted by Lund University. Response preferences are listed by the administrator who according to a follow-up schedule may remind or prompt participants via their personal choice of contact, to fulfil questionnaires and follow-ups. All data will be stored in LUSEC which is an all-inclusive “virtual desk” for storing, managing, and analysing personal and sensitive data that requires high security in compliance with legislation, for example, the EU’s General Data Protection Regulation (GDPR). The platform has a storage solution with encryption of data, a secure user environment with a two-step log-in system using two applications, and a secure file transfer software. Physical material will be stored in secured cupboards in locked fire-safe facilities.

### Statistical methods

The hypotheses tests for the primary and secondary data will be performed on the whole group, and analysis will follow the ITT principle. Data from all sites will be pooled and CI and OR will be estimated for primary outcome (binary), using multi-variable logistic regression models, in line with previous research [29]. Missing data on primary outcome is estimated to be negligible since we use LISA national register data, with below 5% of missingness [79].

Descriptive statistics will be provided to analyse baseline data and help to detect statistically significant differences of interventions group data before allocation. Mixed-effects regression models will be used (secondary outcomes) according to outcome characteristics, time-based (e.g. job-tenure), count-based (e.g. days in employment), binary (e.g. education rate (yes/no), ordinal or longitudinal data if applicable (e.g. health, quality of life and wellbeing outcomes), and proportional-hazard regression (e.g. time to employment) analyses. Repeated measures and time function will be applied in models.

Sensitivity analysis to compare complete data sets and missing data to meet prerequisites for multiple imputations and for co-variety [80]. Sub-analysis will also be performed in relation to intervention site (random), gender, age and alcohol and drug use. Alpha will be set at 5% with associated 95% CIs. Statisticians from the Unit of Medical Statistics and Epidemiology, Clinical Studies Sweden-South Forum, Skånes University Care are consulted to produce a more detailed plan to be published before analysis. Primary data will only be available to researchers in the present trial.

### Monitoring

The PI will have overall responsibility for data and will be supported by the research coordinator who is hired for the trial for independent monitoring and auditing trial conduct. The coordinator will further organise and integrate monitoring with an external trial reference group (meets quarterly), a trial management group (meets biweekly). Additionally, operational work groups will be developed at each site to coproduce any solution for IPS implementation and trial (meets weekly). The Ethics Committee is not part of the auditing trial conduct.

Steps and passages of the trial, e.g. recruitment, enrolment, quality of data, analysis, safety, harms and unintended effects of the interventions or trial conduct will be monitored and communicated. A report of the progression of the trial will be conducted by the trial management group and conclude significant benefits and risks (= 1 year since enrolment). No interim results of data will be performed. The trial steering and management group will involve members from the AS who deliver the intervention, as well as public and user involvement organisations. No data monitoring committee is appointed by the trial sponsor (FORTE) but monitors the trial online via the administrative research system of PRISMA (https://prisma.research.se/. The sponsor requires updates annually, a brief report on the progression of the trial by halftime, while a thorough report of advancements and results is presented post-trial. The PI is responsible for these activities together with the Head of the Department of Health Sciences and the chief economist.

Data and safety measures will further be covered by monitoring groups mentioned and experienced research administrators and statisticians. In addition, unforeseen events and harms (Adverse Events, AE) among participants will be continuously observed and recorded in a special document (AE form) and be instantly reported by the trial management group to the trial steering committee and PI. These events may include suicide attempts and hospitalisation, for example.

### Dissemination plans

Trial results will be disseminated to AS providers, service users, service user organisations and the public by means of peer-reviewed scientific journal publications and popular reports written so that all participants can share the results. Since the present trial is performed in coproduction, any party may share their learning with others. Workshops with stakeholders and service users will also be held if the IPS-ADAS trial is beneficial and further implemented. All scientific publications will have open access and results will further be disseminated in national and international conferences and research networks. Authors will conform to the guidelines of ICMJE (https://www.icmje.org/) when conducting, editing, and publishing in medical journals.

## Discussion

A primary study on the effectiveness of IPS on employment for the new target group and within a new field of practice. Results will further add to the international IPS knowledge base and inform national policy to include an underrepresented group of addictions into working life. To our knowledge, the IPS-ADAS will be the first RCT to investigate the effectiveness of IPS in Sweden. Thus, this study will provide important information about the effectiveness of IPS in employment, health, quality of life and well-being for a new target. It will also deepen our understanding of the complex situation that many of these people find themselves in, a group who find themselves in great need of hope and support to develop a work identity and finding a job. Our partnership with user organisations and AS providers will further provide knowledge and clarity of how coproduction efforts may enhance the implementation of IPS and optimise the quality of the trial and to build capacity and work culture to engage in future research. Coproduction will combine experienced knowledge from all stakeholders, including researchers, and is presumed to reinforce recovery-oriented knowledge into practice that matters for service users.

Positive results could also justify IPS as an evidence-based intervention to be used routinely for persons with addiction in Sweden, and in this case, expected to be used more frequently and be offered as a critical ingredient in future innovations within Addiction service and treatment. To provide opportunities for work and health early in the life course is also a critical shift for proactive ageing [81].

## Trial status

This is the 1.1 protocol of the trial which will be submitted to the BMC Trial on 3 November 2023. The overall start date of the planning and application of Ethical approval was 1 September 2022, and the end date of data collection is planned for 30 June 2026. Recruitment of the first AS site was on 15 January 2023. The start date for recruitment of participants was on 1 September 2023, in tandem with approval and receipt of research funding (FORTE, register number 2023–00080) and registration of the IPS-ADAS trial at the WHO International Clinical Trials Registry Platform on 14 August 2023, with the trial register number ISRCTN10492363. Recruitment will end on 28 February 2025. Updates and modifications of the trial will be updated.

### Supplementary Information

**Supplementary Material 1.**

## Data Availability

The principal investigator and co-authors will be given access to blinded and anonymised data after completion of the trial. Data will be collected by means of the RedCap online system and be stored at LUSEC at Lund University with restricted access. Researchers who are external may get access to data once it is completed, on request, in accordance with the data protection and data exchange regulations agreements. In such circumstances, data will be anonymised and lack information connected to the identity of participants.

## Abbreviations

AS: Addiction Services
IPS: Individual Placement and Support
ITT: Intention to Treat
LUSEC: Lund University SECure virtual desk for storing, managing, and analysing data that requires high security in compliance with legislations
RCT: Randomised Controlled Trial
RedCap: Research Electronic Data Capture
TAU: Treatment as Usual
TVR: Traditional Vocational Rehabilitation
